# Association of one-hour postload plasma glucose combined with metabolic dysfunction-associated steatotic liver disease and FIB-4 screening positivity with elevated LDL cholesterol and cerebrovascular stenosis

**DOI:** 10.3389/fendo.2026.1887611

**Published:** 2026-08-14

**Authors:** Fengtong Zuo, XiaoYan Wang, Changan Xu

**Affiliations:** 1 Department of Cerebrovascular Diseases (Third Division), Cangzhou Central Hospital, Cangzhou, Hebei, China; 2 Department 2 of Neuroimmunology and Central Nervous System Infection, Cangzhou Central Hospital, Cangzhou, Hebei, China; 3 Department of Endocrinology, Tianjin People’s Hospital, Tianjin, China

**Keywords:** 1-hour postload plasma glucose, cross-sectional analysis, fibrosis-4 index, metabolic dysfunction-associated steatotic liver disease, type 2 diabetes mellitus

## Abstract

**Background:**

Metabolic dysfunction-associated steatotic liver disease (MASLD) is highly prevalent in type 2 diabetes mellitus (T2DM) and amplifies cardiovascular and metabolic burden. Elevated low-density lipoprotein cholesterol (LDL-C) is a key driver of atherosclerotic cerebrovascular stenosis, and MASLD-mediated hepatic lipid dysregulation is closely linked to LDL-C metabolism. One-hour postload plasma glucose (1h-PG) reflects early beta-cell dysfunction and may synergistically contribute to cerebrovascular injury alongside the MASLD/FIB-4 phenotype.

**Methods:**

This single-center retrospective cross-sectional study enrolled 312 treatment-naïve adults with newly diagnosed T2DM, of whom 278 completed a standardized 75-g oral glucose tolerance test (OGTT; age 24–78 years). The primary outcome was ultrasound-confirmed MASLD combined with age-specific FIB-4 screening positivity (age <65 years: FIB-4 ≥1.30; age ≥65 years: FIB-4 ≥2.00). Secondary outcomes included elevated LDL-C (≥3.4 mmol/L) and cerebrovascular stenosis (≥50% luminal narrowing in major intracranial or extracranial arteries).

**Results:**

Overall, 199 participants (71.6%) had MASLD and 64 (23.0%) met the primary outcome. Those meeting the primary outcome had significantly higher 1h-PG (17.7 ± 3.7 vs. 14.9 ± 3.8 mmol/L; P<0.001), with an adjusted OR of 1.15 per 1-mmol/L increment (95% CI 1.05–1.26; P = 0.003). Elevated LDL-C was present in 73 participants (26.3%) and cerebrovascular stenosis in 49 (17.6%). The composite group of high 1h-PG (≥Q3) plus MASLD/FIB-4 positivity had the highest stenosis rate (34.8% vs. 6.0%), with an adjusted OR of 2.61 (95% CI 1.23–5.55; P = 0.012). Adding LDL-C attenuated the OR from 3.42 to 2.61 (~24%), indicating partial mediation. The AUC improved from 0.661 (1h-PG alone) to 0.726 (combined model).

**Conclusions:**

In newly diagnosed T2DM, elevated 1h-PG was associated with MASLD/FIB-4 positivity; their combination was further linked to higher LDL-C and increased cerebrovascular stenosis risk, with LDL-C potentially acting as a partial mediator. The combined model showed improved discriminative ability, though validation in larger multicenter cohorts remains warranted.

## 1 Introduction

Metabolic dysfunction-associated steatotic liver disease (MASLD) is defined by the presence of hepatic steatosis accompanied by at least one cardiometabolic risk factor; current nomenclature emphasizes the link between metabolic abnormalities and the hepatic phenotype (1). Type 2 diabetes mellitus (T2DM) and MASLD are highly comorbid, and together they increase the burden of liver-related and cardiovascular adverse outcomes (2). In patients with T2DM, a hepatic phenotype with greater disease activity and higher fibrosis probability is not uncommon, making systematic non-invasive risk stratification clinically necessary (3).

The fibrosis burden in MASLD is closely related to long-term outcomes, and changes in non-invasive fibrosis markers and elastography provide important prognostic information (4). The Fibrosis-4 (FIB-4) index, calculated from age, aspartate aminotransferase (AST), alanine aminotransferase (ALT), and platelet count, is used primarily as a first-line exclusion screening tool and for identifying individuals requiring further evaluation (5). The pathophysiology of MASLD involves interactions among insulin resistance, lipotoxicity, oxidative stress, inflammation, and pro-fibrotic pathways (6).

One-hour postload plasma glucose (1h-PG) reflects early beta-cell dysfunction and abnormal glucose disposal and provides dynamic glycemic information distinct from fasting indices (7). Elevated 1h-PG during the oral glucose tolerance test (OGTT) is associated with impaired beta-cell function and subsequent deterioration of glucose metabolism, supporting the value of this time-point in distinguishing metabolic phenotypes (8). Postprandial glucose is an important component of the glycemic continuum and can complement fasting plasma glucose (FPG) and glycated hemoglobin (HbA1c) in characterizing dynamic metabolic status (9).

Elevated LDL-C is a core risk factor for atherosclerosis, and MASLD-mediated hepatic lipid metabolic disruption—including overproduction of very-low-density lipoprotein (VLDL) and downregulation of LDL receptors—can directly increase circulating LDL-C levels (7). In patients with T2DM, insulin resistance and hyperglycemia synergistically promote the formation of small dense LDL particles and LDL oxidative modification, accelerating atherosclerosis (8). Cerebrovascular stenosis, particularly intracranial and internal carotid artery stenosis, occurs significantly more frequently in T2DM patients than in the general population; it is a major pathological basis of ischemic stroke and is closely associated with metabolic syndrome components including MASLD, hyperglycemia, and dyslipidemia (9). The acute postprandial glycemic stress reflected by 1h-PG may injure vascular endothelium and promote LDL oxidation and glycation through multiple pathways, thereby establishing a potential pathological link between metabolic dysfunction and cerebrovascular stenosis (10). However, whether 1h-PG combined with MASLD/FIB-4 can synergistically predict elevated LDL-C and cerebrovascular stenosis has not been systematically evaluated in newly diagnosed, treatment-naïve T2DM populations.

Existing evidence suggests that dynamic glycemic exposure is associated with MASLD severity in T2DM patients, but the combined relationship of 1h-PG with age-specific FIB-4 screening phenotypes, elevated LDL-C, and cerebrovascular stenosis has not been systematically evaluated in newly diagnosed patients who are treatment-naïve before the loading test (11). Using a standardized 75-g OGTT cohort as the primary analysis population, this study evaluated: (1) the association between 1h-PG and MASLD combined with FIB-4 screening positivity; (2) the predictive value of the combined 1h-PG and MASLD/FIB-4 composite index for elevated LDL-C and cerebrovascular stenosis; and (3) the potential mediating role of LDL-C between these metabolic markers and cerebrovascular injury.

## 2 Materials and methods

### 2.1 Study design and participants

This was a single-center retrospective cross-sectional analysis. Participants were adults with newly diagnosed T2DM who completed an initial systematic evaluation at the Endocrinology Department of our institution between January 2021 and December 2025. Exposures, outcomes, and covariates were all derived from the same initial assessment window. T2DM was diagnosed based on HbA1c, fasting plasma glucose, 2-hour plasma glucose during a 75-g OGTT, or random plasma glucose with typical symptoms (11).

This study was reported in accordance with the Strengthening the Reporting of Observational Studies in Epidemiology (STROBE) statement (12). Newly diagnosed T2DM was defined as no prior history of diabetes, first-time fulfillment of diagnostic criteria, and an interval from diagnosis to systematic evaluation of no more than 3 months. Inclusion criteria were age ≥18 years, completion of a 75-g OGTT or standardized meal challenge with a venous plasma glucose result at 60 ± 10 minutes, concurrent liver ultrasound, and pre-specified laboratory measurements.

Exclusion criteria included other types of diabetes, pre-existing chronic liver disease, alcohol consumption above pre-specified thresholds, recent use of medications affecting liver fat or glucose metabolism, severe acute or chronic illness, pregnancy or lactation, antidiabetic treatment before the loading test, missing essential variables, or a 60-minute sample outside the allowable time window. The study protocol was approved by the Institutional Ethics Committee, with individual informed consent waived.

### 2.2 Clinical, laboratory, and loading test data

Demographic characteristics, medical history, medication use, smoking and drinking status, height, weight, waist circumference, and blood pressure were recorded at the initial systematic evaluation. Body mass index (BMI) was calculated as weight (kg) divided by height squared (m²). Hypertension was defined as a previous diagnosis, current use of antihypertensive medication, or repeated measurements on separate days showing systolic blood pressure ≥140 mmHg and/or diastolic blood pressure ≥90 mmHg.

Morning fasting venous blood was used to measure FPG, HbA1c, insulin, C-peptide, total cholesterol (TC), triglycerides (TG), high-density lipoprotein cholesterol (HDL-C), LDL-C, ALT, AST, γ-glutamyltransferase (GGT), albumin, total bilirubin, platelet count, creatinine, and uric acid. Plasma glucose was measured by the hexokinase method, and HbA1c was measured by high-performance liquid chromatography. Estimated glomerular filtration rate (eGFR) was calculated using the 2021 Chronic Kidney Disease Epidemiology Collaboration (CKD-EPI) creatinine equation (13).

The primary exposure was venous plasma glucose measured 60 minutes after the 75-g OGTT (1h-PG). After fasting for 8–10 hours, participants drank 75 g of anhydrous glucose dissolved in 250–300 mL of water within 5 minutes. Venous blood was collected at 0, 60, and 120 minutes; timing began when participants started drinking the glucose solution. Participants remained seated during the test and refrained from eating, smoking, and unnecessary activity. The 60-minute sample was allowed within a ±10-minute window.

Standardized meal challenge data were used for an independent supplementary analysis: the standardized meal provided 500 kcal, comprising 70 g carbohydrates, 18 g protein, and 16.5 g fat. Participants completed the meal within 15 minutes under nurse supervision; timing began from the first bite, and venous blood was collected at 60 ± 10 minutes.

### 2.3 MASLD and FIB-4 screening outcomes

MASLD was defined by the presence of hepatic steatosis evidence and at least one cardiometabolic risk factor; since all participants in this study had T2DM, all met the metabolic risk factor criterion (14).

Hepatic steatosis was assessed by qualified radiologists based on enhanced hepatic parenchymal echogenicity, increased hepatorenal echo contrast, posterior acoustic attenuation, and obscured intrahepatic vascular structures. Formal imaging reports served as the data source. Ultrasound reports did not provide quantitative hepatic fat content (15).

FIB-4 was calculated as: age (years) × AST (U/L)/[platelet count (10^9^/L) × √ALT (U/L)]. The primary outcome was ultrasound-diagnosed MASLD combined with elevated first-line FIB-4 screening results, using age-specific thresholds: FIB-4 ≥1.30 for age <65 years and FIB-4 ≥2.00 for age ≥65 years. Fixed thresholds of FIB-4 ≥1.30 and FIB-4 >2.67 were used in sensitivity analyses and for sparse-event outcome analyses, respectively. Elevated screening results identify individuals requiring further non-invasive evaluation (16).

Because FIB-4 discriminative performance is age-dependent, this study reported the distribution of participants aged <35 years and ≥65 years, and conducted analyses excluding participants aged <35 years and age-stratified analyses, to examine the impact of age structure on association estimates (17).

MASLD patients were classified into three categories: age-specific low-risk, intermediate-risk, and high-risk (FIB-4 >2.67). For participants aged <65 years, low risk was defined as FIB-4 <1.30, and intermediate risk as 1.30–2.67; for participants aged ≥65 years, low risk was FIB-4 <2.00, and intermediate risk was 2.00–2.67. FIB-4 >2.67 defined high risk across all age groups. Transient elastography, magnetic resonance elastography (MRE), Enhanced Liver Fibrosis (ELF) testing, or liver histology were not available in this dataset (18). Secondary outcomes included elevated LDL-C (defined as LDL-C ≥3.4 mmol/L, corresponding to the borderline high threshold of the Chinese Guidelines for the Prevention and Treatment of Dyslipidemia in Adults).

### 2.4 Missing data and statistical analysis

**Figure 1:**
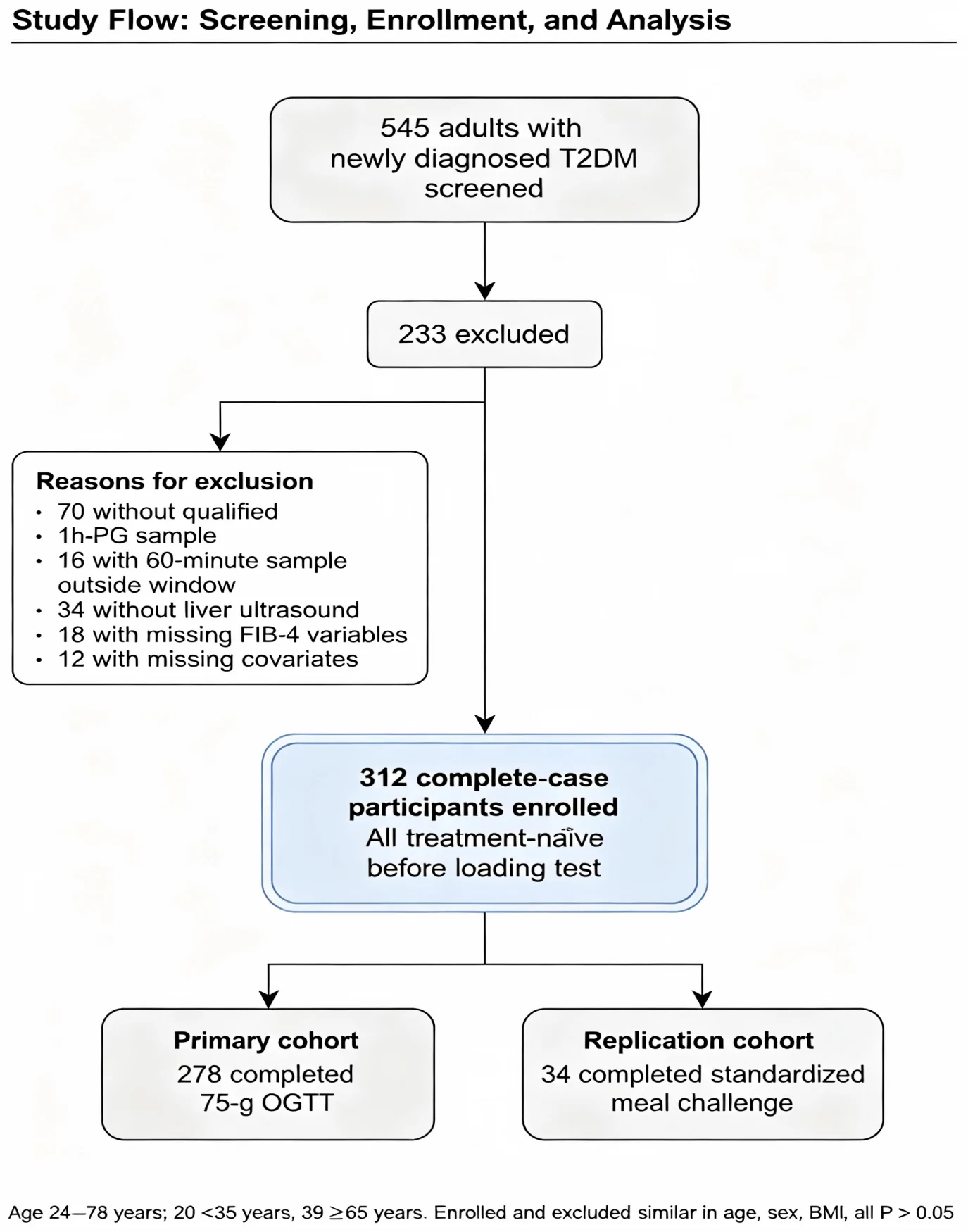
Participant flow diagram (CONSORT-style). Exclusion reasons are counted by the first applicable criterion (mutually exclusive). The final complete-case analytic set comprised 312 participants, of whom 278 constituted the primary 75-g OGTT cohort and 34 the supplementary standardized-meal cohort. The primary screening outcome was defined as ultrasound-diagnosed MASLD combined with a FIB-4 index meeting the age-specific threshold. 1h-PG, 1-hour postload plasma glucose; FIB-4, Fibrosis-4 index; MASLD, metabolic dysfunction-associated steatotic liver disease; OGTT, oral glucose tolerance test; T2DM, type 2 diabetes mellitus. Age-specific FIB-4 thresholds: ≥1.30 (age <65 years); ≥2.00 (age ≥65 years).

Missing data for 1h-PG, liver ultrasound, FIB-4 component variables, and pre-specified covariates were tabulated in a listing. According to pre-specified protocols, participants with missing exposure, ultrasound, or FIB-4 component variables, and those with 60-minute samples outside the ±10-minute window were excluded. Participants with missing pre-specified covariates were handled by complete-case analysis without imputation. Exclusion reasons and missing counts are presented in **Figure 1** and **Supplementary Table 1**.

Normally distributed continuous variables are expressed as mean ± standard deviation (SD); independent-samples t-test was used when variances were equal, and Welch t-test when unequal. Non-normally distributed variables are expressed as median [interquartile range (IQR)] and compared using the Mann–Whitney U test. Categorical variables are expressed as number (percentage) and compared using χ² or Fisher’s exact test. Correlations were assessed using Pearson or Spearman rank correlation coefficients according to variable distribution.

The proportion of outcomes across 1h-PG quartiles was compared by χ² test, and the Cochran–Armitage test was used to assess ordinal trends. Continuous 1h-PG was the primary effect form; quartiles were used only to describe the dose gradient.

The primary multivariable logistic regression model was pre-specified to include 1h-PG, age, sex, BMI, hypertension, HbA1c, log_2_-transformed TG, HDL-C, and eGFR (9 parameters total). Adjusted odds ratios (ORs) and 95% confidence intervals (CIs) are reported. Covariates were pre-specified based on clinical knowledge and their potential relationships with 1h-PG and the MASLD/FIB-4 screening phenotype, not selected based on univariable P-values or between-group differences.

A parallel 8-parameter sensitivity model excluding all FIB-4 component variables was also fitted. Parallel models added 1h-PG, 2-hour postload plasma glucose (2h-PG), FPG, or HbA1c to the same age-adjusted non-glycemic baseline model and reported OR, P-value, Akaike information criterion (AIC), area under the receiver operating characteristic curve (AUC), Brier score, and calibration slope. Nested models were compared by likelihood-ratio tests.

Optimism bias, calibration slope, and uniform shrinkage factor were estimated using 1,000 bootstrap resamples. Restricted cubic spline knots were placed at the 10th, 50th, and 90th percentiles. The FIB-4 >2.67 endpoint was analyzed using a pre-specified 5-parameter Firth logistic regression model. Exploratory ROC candidate cut-offs were determined by the Youden index. The 95% CIs for sensitivity, specificity, positive predictive value (PPV), and negative predictive value (NPV) were calculated using the Wilson method. All tests were two-sided with α = 0.05. Statistical analyses were performed using R 4.3.2 and SPSS 26.0.

For secondary outcomes (elevated LDL-C and cerebrovascular stenosis), binary logistic regression was used to assess the associations of 1h-PG, MASLD/FIB-4 screening status, and their composite index with the outcomes, adjusting for the same covariates as the primary analysis. The composite index was defined as co-presence of 1h-PG ≥Q3 quartile (15.26 mmol/L) and MASLD/FIB-4 screening positivity. Between-group differences in LDL-C were assessed by independent-samples t-test or Welch t-test. The mediating effect of LDL-C on the association between the composite index and cerebrovascular stenosis was assessed by stepwise regression (≥10% change in OR after adding LDL-C was considered substantial mediation). Exploratory AUC comparisons for cerebrovascular stenosis were performed using the DeLong method.

### 2.5 Cerebrovascular stenosis assessment

At the initial systematic evaluation, all participants underwent carotid vascular ultrasound performed by qualified sonographers to assess luminal stenosis of the bilateral common carotid arteries, internal carotid arteries, and external carotid arteries; participants with clinical indications (including neurological symptoms, carotid bruit, or multiple cerebrovascular risk factors) additionally underwent transcranial Doppler (TCD) to assess the major intracranial arteries (middle cerebral artery, anterior cerebral artery, posterior cerebral artery, and vertebrobasilar arteries). Cerebrovascular stenosis was defined as luminal diameter stenosis ≥50% in any of these arteries. Stenosis degree was calculated by the NASCET method, with formal imaging reports serving as the data source. Magnetic resonance angiography (MRA) or computed tomography angiography (CTA) data were not included; mild stenosis (<50%) and diffuse intimal thickening were recorded but not included in the outcome definition. Cerebrovascular assessment was completed within the same assessment window as liver ultrasound and laboratory testing, with assessors blinded to participants’ glycemic and hepatic status.

## 3 Results

### 3.1 Participant flow and population characteristics

During the study period, 545 adults with newly diagnosed T2DM were screened; 233 were excluded by the first applicable reason, and 312 complete-case participants were ultimately enrolled. Of these, 278 completed the 75-g OGTT (primary cohort) and 34 completed the standardized meal challenge. Major missing or disqualified items included 70 participants without a qualified 1h-PG sample, 16 with 60-minute samples outside the allowable window, 34 without concurrent liver ultrasound, 18 with missing FIB-4 component variables, and 12 with missing pre-specified covariates. The age range was 24–78 years; 20 participants were aged <35 years and 39 were aged ≥65 years. All participants in the final analysis were treatment-naïve before the loading test. Enrolled and excluded participants did not differ significantly in available age, sex, or BMI (all P > 0.05) (**Figure 1**; **Supplementary Tables 1**, **2**). Cerebrovascular assessment was completed in all 312 enrolled participants; all 278 in the primary OGTT cohort had qualified carotid ultrasound reports, and 129 (46.4%) additionally underwent TCD due to clinical indications.

Participants meeting the primary screening outcome had descriptively higher age, lower male proportion and height, and higher BMI, waist circumference, diastolic blood pressure, hypertension prevalence, and antihypertensive medication use (all P < 0.05). Time from diagnosis to evaluation, smoking history, alcohol use, coronary artery disease history, lipid-lowering medication use, body weight, and systolic blood pressure did not differ significantly (all P > 0.05) (**Table 1**).

**Table 1:** Baseline clinical characteristics stratified by MASLD combined with FIB-4 screening status defined by age-specific thresholds.

Variable	Total (n=278)	Did not meet primary outcome (n=214)	Met primary outcome (n=64)	Statistical method	P value
Age, years	51.4 ± 10.5	48.9 ± 9.8	59.7 ± 7.7	Descriptive	—
Age range, years	24–78	24–78	41–78	Descriptive	—
Age <35 years	17 (6.1)	17 (7.9)	0 (0.0)	Descriptive	—
Age ≥65 years	34 (12.2)	16 (7.5)	18 (28.1)	Descriptive	—
Male sex	173 (62.2)	143 (66.8)	30 (46.9)	χ²	0.004
Time from diagnosis to evaluation, days	13.6 [7.7, 23.3]	13.4 [7.6, 22.8]	14.0 [8.1, 25.2]	Mann–Whitney U	0.268
Smoking history	77 (27.7)	58 (27.1)	19 (29.7)	χ²	0.685
Alcohol use below threshold	55 (19.8)	42 (19.6)	13 (20.3)	χ²	0.904
Hypertension	111 (39.9)	72 (33.6)	39 (60.9)	χ²	<0.001
History of CAD	14 (5.0)	8 (3.7)	6 (9.4)	Fisher’s exact	0.098
Antihypertensive medication	79 (28.4)	50 (23.4)	29 (45.3)	χ²	0.001
Lipid-lowering medication	22 (7.9)	17 (7.9)	5 (7.8)	χ²	0.973
Height, m	1.66 ± 0.08	1.67 ± 0.08	1.64 ± 0.08	t-test	0.009
Body weight, kg	74.8 ± 11.3	74.5 ± 11.1	75.6 ± 11.8	t-test	0.494
BMI, kg/m²	27.1 ± 3.3	26.8 ± 3.2	28.1 ± 3.3	t-test	0.005
Waist circumference, cm	93.3 ± 7.5	92.5 ± 7.0	96.0 ± 8.5	Welch t-test	0.003
SBP, mmHg	132.5 ± 15.0	131.5 ± 14.4	136.0 ± 16.5	Welch t-test	0.052
DBP, mmHg	82.3 ± 9.6	81.3 ± 9.0	85.7 ± 10.5	Welch t-test	0.003

BMI, body mass index; CAD, coronary artery disease; DBP, diastolic blood pressure; FIB-4, Fibrosis-4 index; MASLD, metabolic dysfunction-associated steatotic liver disease; SBP, systolic blood pressure. Values are mean ± SD, median [IQR], or n (%). P values are two-sided.

Participants meeting the primary screening outcome had higher FPG, 1h-PG, 2h-PG, HbA1c, fasting insulin, fasting C-peptide, TG, and GGT, and lower HDL-C, albumin, and eGFR (all P < 0.05). TC, LDL-C, total bilirubin, creatinine, and uric acid did not differ significantly (all P > 0.05). ALT, AST, platelet count, MASLD status, FIB-4, and FIB-4 >2.67 constitute the outcome definition or are structurally related to it, and are therefore presented descriptively only (**Table 2**). 1h-PG showed strong positive correlation with 2h-PG, weak to moderate positive correlations with FIB-4, FPG, HbA1c, and AST, and weak negative correlation with platelet count; its correlations with BMI and TG were not statistically significant (significant correlations P < 0.05; others P > 0.05) (**Supplementary Table 3**).

**Table 2:** Laboratory and liver-related variables stratified by MASLD combined with FIB-4 screening status defined by age-specific thresholds.

Variable	Total (n=278)	Did not meet primary outcome (n=214)	Met primary outcome (n=64)	Statistical method	P value
FPG, mmol/L	9.4 ± 1.9	9.2 ± 1.9	10.0 ± 2.0	t-test	0.004
1h-PG, mmol/L	15.5 ± 4.0	14.9 ± 3.8	17.7 ± 3.7	Welch t-test	<0.001
2h-PG, mmol/L	13.4 ± 2.9	13.0 ± 2.7	14.9 ± 2.9	t-test	<0.001
HbA1c, %	8.7 ± 1.6	8.6 ± 1.5	9.2 ± 1.7	Welch t-test	0.013
Fasting insulin, mIU/L	12.5 [9.0, 18.2]	12.0 [8.8, 17.7]	14.8 [10.1, 19.0]	Mann–Whitney U	0.012
Fasting C-peptide, ng/mL	2.7 ± 0.9	2.6 ± 0.9	2.9 ± 0.8	t-test	0.017
TC, mmol/L	5.0 ± 1.0	5.0 ± 1.0	5.1 ± 1.0	t-test	0.483
TG, mmol/L	1.8 [1.3, 2.7]	1.8 [1.3, 2.6]	2.0 [1.4, 3.4]	Mann–Whitney U	0.041
HDL-C, mmol/L	1.08 ± 0.26	1.10 ± 0.27	1.00 ± 0.22	Welch t-test	0.003
LDL-C, mmol/L	2.91 ± 0.81	2.89 ± 0.82	2.98 ± 0.78	t-test	0.437
ALT, U/L	37.2 [27.2, 50.8]	35.1 [26.5, 46.5]	45.2 [33.6, 63.0]	Descriptive	—
AST, U/L	28.6 [20.3, 38.5]	25.6 [18.6, 33.5]	39.0 [29.5, 53.2]	Descriptive	—
GGT, U/L	42.3 [29.0, 63.5]	38.2 [27.2, 55.8]	55.3 [40.1, 81.0]	Mann–Whitney U	<0.001
Albumin, g/L	43.6 ± 3.1	43.8 ± 3.2	42.9 ± 2.9	t-test	0.045
Total bilirubin, μmol/L	12.7 [9.9, 16.1]	12.3 [9.6, 16.0]	13.8 [10.7, 16.8]	Mann–Whitney U	0.112
Platelet count, ×10^9^/L	228.7 ± 51.0	243.0 ± 45.3	181.0 ± 39.5	Descriptive	—
Creatinine, μmol/L	71.3 ± 13.2	70.7 ± 13.1	73.1 ± 13.5	t-test	0.203
Uric acid, μmol/L	350.9 ± 79.7	347.0 ± 80.0	364.0 ± 77.8	t-test	0.135
eGFR, mL/min/1.73 m²	105.7 ± 11.5	107.5 ± 10.8	99.6 ± 11.0	t-test	<0.001
Ultrasound-diagnosed MASLD	199 (71.6)	135 (63.1)	64 (100.0)	Descriptive	—
FIB-4	0.96 [0.77, 1.42]	0.86 [0.72, 1.01]	1.86 [1.55, 2.58]	Descriptive	—
FIB-4 >2.67	16 (5.8)	0 (0.0)	16 (25.0)	Descriptive	—

LDL-C did not differ significantly between primary MASLD/FIB-4 screening outcome groups (P = 0.437), but was significantly different between cerebrovascular stenosis groups (3.38 ± 0.74 vs 2.79 ± 0.80 mmol/L; P < 0.001; see Section 3.9). ALT, alanine aminotransferase; AST, aspartate aminotransferase; eGFR, estimated glomerular filtration rate; FIB-4, Fibrosis-4 index; FPG, fasting plasma glucose; GGT, γ-glutamyltransferase; HbA1c, glycated hemoglobin; HDL-C, high-density lipoprotein cholesterol; LDL-C, low-density lipoprotein cholesterol; MASLD, metabolic dysfunction-associated steatotic liver disease; TC, total cholesterol; TG, triglycerides; 1h-PG, one-hour postload plasma glucose; 2h-PG, two-hour postload plasma glucose.

### 3.2 Age distribution and FIB-4 screening classification

Among all 312 participants, 224 had MASLD and 72 met the MASLD combined with age-specific FIB-4 screening positivity. In the primary OGTT cohort, the corresponding numbers were 199 and 64. Participants aged <35, 35–64, and ≥65 years numbered 20, 253, and 39, respectively, with 0, 51, and 21 meeting the primary screening outcome. Among all MASLD patients, the age-specific low-risk, intermediate-risk, and high-risk (FIB-4 >2.67) categories included 152, 52, and 20 participants, respectively. The OGTT-MASLD subgroup had 135, 48, and 16, and the standardized-meal MASLD subgroup had 17, 4, and 4 (**Table 3**). As a sensitivity outcome, MASLD combined with elevated FIB-4 by the fixed threshold ≥1.30 was present in 84 of 312 participants (75/278 in the primary OGTT cohort; 9/34 in the standardized-meal cohort).

**Table 3:** Age distribution and three-tier FIB-4 grading in MASLD patients.

Population/age stratum	Total	75-g OGTT	Standardized meal	FIB-4 screening positive (age-specific threshold)
Age <35 years	20	17	3	0
Age 35–64 years	253	227	26	51
Age ≥65 years	39	34	5	21
Total	312	278	34	72
All MASLD: age-specific low-risk	152 (67.9%)	135 (67.8%)	17 (68.0%)	—
All MASLD: intermediate-risk	52 (23.2%)	48 (24.1%)	4 (16.0%)	52
All MASLD: FIB-4 >2.67	20 (8.9%)	16 (8.0%)	4 (16.0%)	20

MASLD, metabolic dysfunction-associated steatotic liver disease; FIB-4, Fibrosis-4 index; OGTT, oral glucose tolerance test. Percentages use the number of MASLD patients within each loading test type as denominator. — = not applicable.

### 3.3 1h-PG quartile distribution

**Figure 2:**
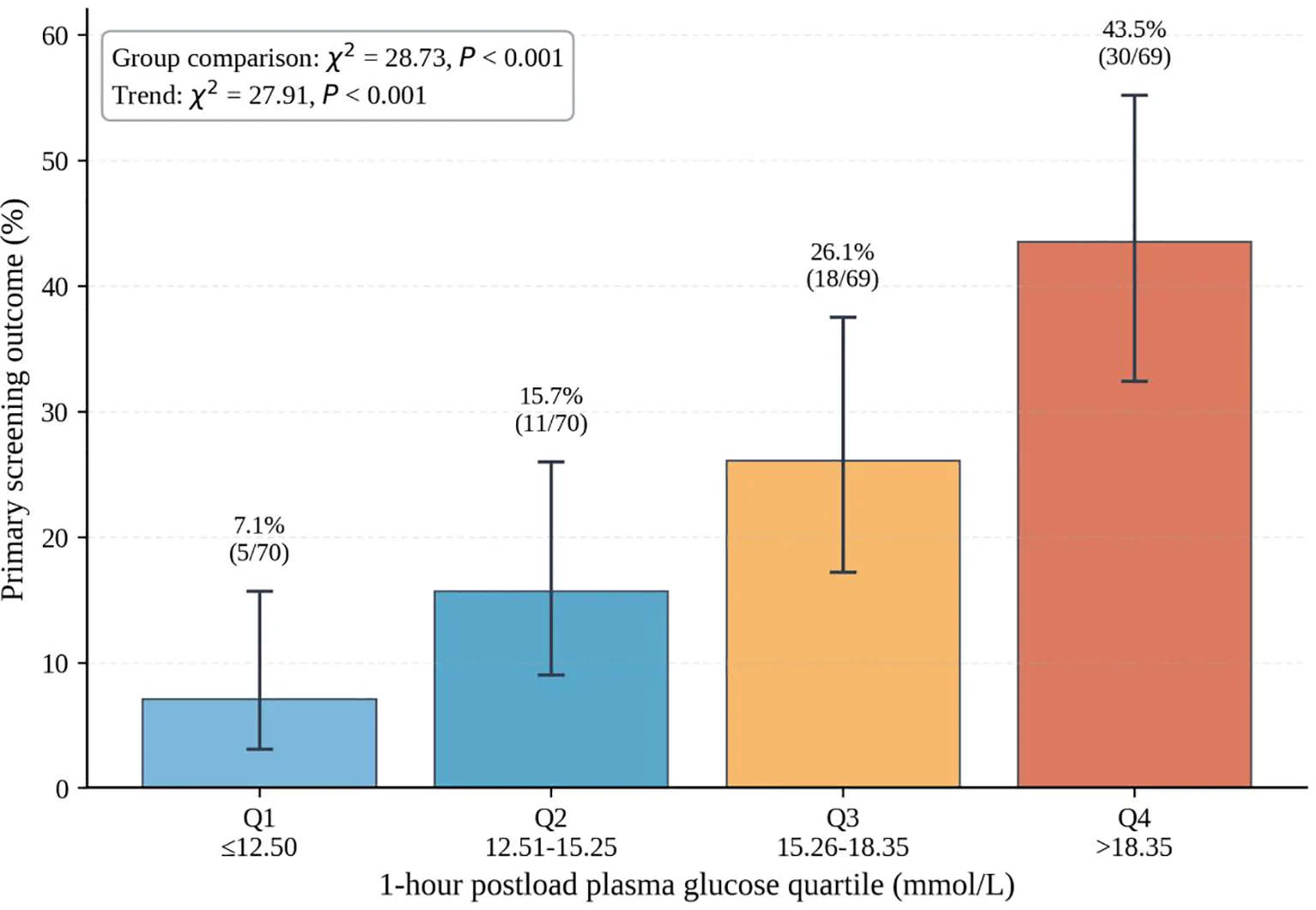
Proportions of the primary screening outcome across one-hour postload plasma glucose (1h-PG) quartiles. Q1–Q4 denote quartiles defined from the lowest to highest 1h-PG distribution. Bar heights indicate proportions; error bars indicate binomial Wilson 95% confidence intervals. Between-group χ² = 28.73; Cochran–Armitage trend χ² = 27.91; both P < 0.001. The primary screening outcome was defined as ultrasound-diagnosed MASLD combined with a FIB-4 index meeting the age-specific threshold.

As 1h-PG quartiles increased from Q1 (lowest) to Q4 (highest), the proportion of participants meeting the primary screening outcome progressively increased, with statistically significant between-group differences and ordinal trends (both P < 0.001) (**Supplementary Table 4**; **Figure 2**).

### 3.4 Primary multivariable model

The primary model included 64 outcome events and 9 parameters (events-per-variable ratio = 7.1). The adjusted OR per 1-mmol/L increment of 1h-PG was 1.15 (95% CI 1.05–1.26; P = 0.003), and the adjusted OR per 1-SD increment (4.00 mmol/L) was 1.73 (95% CI 1.20–2.50; P = 0.003). Age was associated with the primary screening outcome (P < 0.001); all other covariates were non-significant (all P > 0.05) (**Table 4**).

**Table 4:** Full primary model for the association between 1h-PG and MASLD combined with FIB-4 screening positivity defined by age-specific thresholds.

Variable	β	SE	OR (95% CI)	Wald χ²	P value
1h-PG, per SD (4.00 mmol/L)	0.548	0.187	1.73 (1.20–2.50)	8.59	0.003
Age, per 5 years	0.507	0.100	1.66 (1.36–2.02)	25.70	<0.001
Male sex	−0.300	0.340	0.74 (0.38–1.44)	0.78	0.378
BMI, per 1 kg/m²	0.067	0.047	1.07 (0.98–1.17)	2.03	0.154
Hypertension	0.350	0.342	1.42 (0.73–2.77)	1.05	0.306
HbA1c, per 1%	0.095	0.093	1.10 (0.92–1.32)	1.04	0.307
TG, per doubling	0.150	0.195	1.16 (0.79–1.70)	0.59	0.442
HDL-C, per 0.1 mmol/L	−0.050	0.055	0.95 (0.85–1.06)	0.83	0.363
eGFR, per 10 mL/min/1.73 m²	−0.130	0.136	0.88 (0.68–1.15)	0.91	0.339

n = 278; 64 outcome events. BMI, body mass index; eGFR, estimated glomerular filtration rate; HbA1c, glycated hemoglobin; HDL-C, high-density lipoprotein cholesterol; MASLD, metabolic dysfunction-associated steatotic liver disease; OR, odds ratio; SD, standard deviation; SE, standard error; TG, triglycerides; 1h-PG, one-hour postload plasma glucose.

The maximum variance inflation factor (VIF) was 1.88; the apparent AUC was 0.801, the bootstrap optimism-corrected AUC was 0.786, the calibration slope was 0.91, and the shrinkage-adjusted 1h-PG OR was approximately 1.65 (**Supplementary Table 5**).

### 3.5 Parallel models for conventional glycemic markers

The age-adjusted non-glycemic baseline model M0 had an apparent/optimism-corrected AUC of 0.772/0.758. For glycemic marker regression coefficients, the HbA1c model M1, 1h-PG model M2, and 2h-PG model M3 were all associated with the primary screening outcome (P = 0.049, 0.002, and 0.005, respectively), whereas the FPG model M4 did not reach statistical significance (P = 0.095). Adding 1h-PG (M5) and 2h-PG (M6) to M1 both improved model fit (likelihood-ratio test P = 0.004 and 0.009, respectively), whereas adding FPG (M7) did not (likelihood-ratio χ² = 1.30; P = 0.254). The 1h-PG and 2h-PG models generally had lower AIC and Brier scores, higher AUC, and calibration slopes of 0.90–0.92; the AUC difference between M5 and M6 was not statistically significant (P = 0.675) (**Table 5**).

**Table 5:** Comparison of pre-specified parallel glycemic marker models.

Model	Composition	OR per SD (95% CI)	P value	AIC	Apparent/corrected AUC	Brier score	Calibration slope
M0	Age-adjusted non-glycemic baseline	—	—	259.5	0.772/0.758	0.162	0.92
M1	M0 + HbA1c	1.31 (1.00–1.70)	0.049	257.4	0.781/0.767	0.159	0.92
M2	M0 + 1h-PG	1.79 (1.25–2.57)	0.002	250.8	0.797/0.783	0.153	0.91
M3	M0 + 2h-PG	1.67 (1.17–2.39)	0.005	252.1	0.792/0.778	0.155	0.91
M4	M0 + FPG	1.26 (0.96–1.65)	0.095	258.6	0.778/0.764	0.160	0.92
M5	M1 + 1h-PG	1.73 (1.20–2.50)	0.003	251.2	0.801/0.786	0.151	0.91
M6	M1 + 2h-PG	1.56 (1.08–2.25)	0.018	252.5	0.798/0.783	0.153	0.90
M7	M1 + FPG	1.14 (0.85–1.52)	0.383	258.1	0.783/0.768	0.158	0.91

All models used the same primary cohort (n = 278; 64 events). Nested models compared by likelihood-ratio tests: M5 vs M1, χ² = 8.20, P = 0.004; M6 vs M1, χ² = 6.90, P = 0.009; M7 vs M1, χ² = 1.30, P = 0.254. DeLong method AUC comparison of M5 vs M6: difference 0.003 (95% CI −0.011 to 0.018; P = 0.675). AIC, Akaike information criterion; AUC, area under the curve; FPG, fasting plasma glucose; HbA1c, glycated hemoglobin; 1h-PG, one-hour postload plasma glucose; 2h-PG, two-hour postload plasma glucose.

### 3.6 Restricted cubic splines

**Figure 3:**
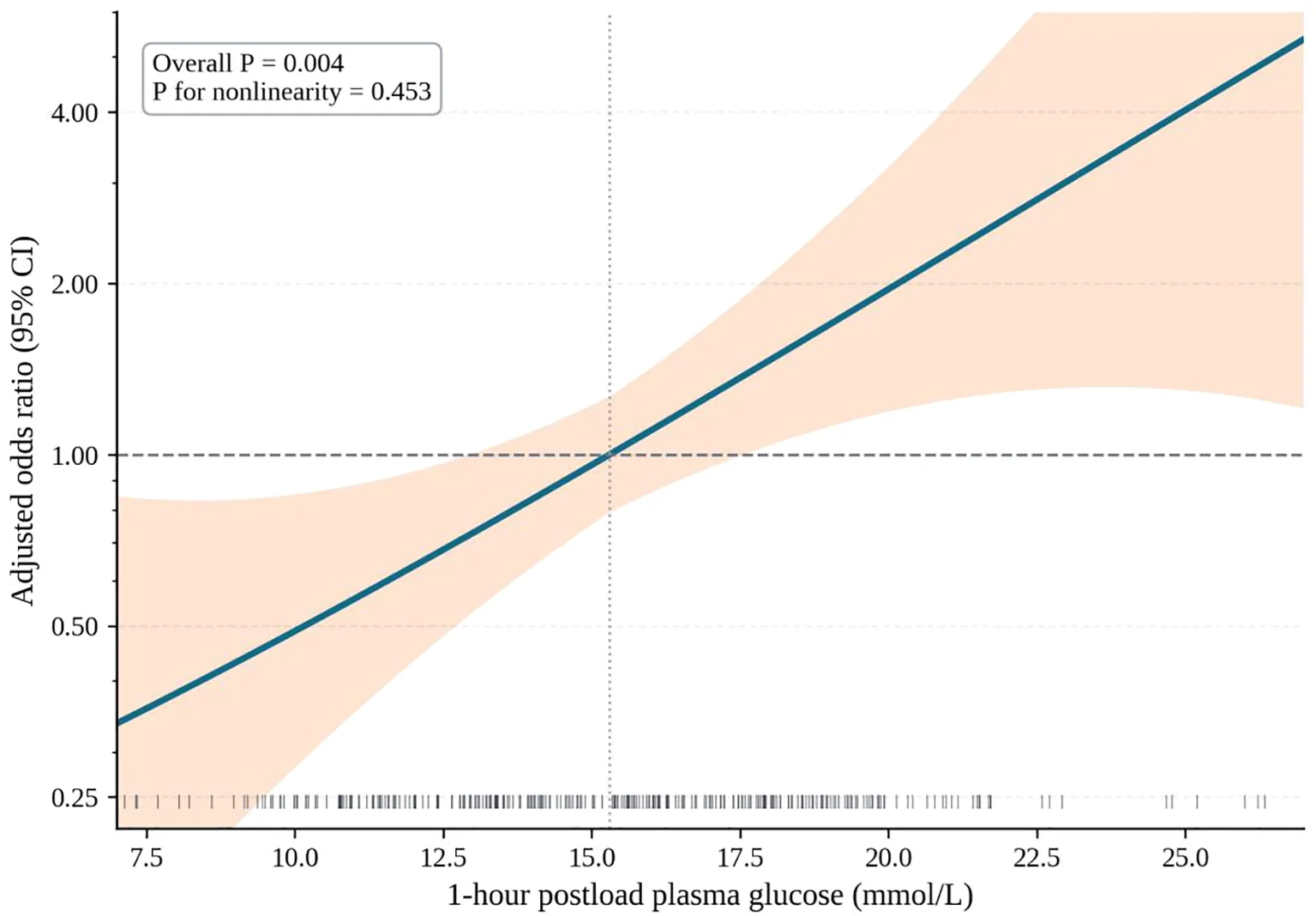
Restricted cubic spline for the association between one-hour postload plasma glucose (1h-PG) and the primary screening outcome. The solid line represents the adjusted odds ratio (OR) after adjustment for age and other pre-specified covariates; the shaded area represents the 95% confidence interval (CI). The y-axis uses a logarithmic scale. The vertical dashed line indicates the median 1h-PG of 15.30 mmol/L, set as the reference (OR = 1). The rug plot at the bottom shows the observed value distribution. Overall P = 0.004; non-linearity P = 0.453, indicating compatibility with an approximately linear association within the studied range.

The restricted cubic spline showed an overall statistically significant association (P = 0.004), while the non-linearity test did not reach statistical significance (P = 0.453), suggesting that the data within the studied range are compatible with an approximately linear association. At the upper tail of the distribution, confidence intervals widened, and the rug plot showed fewer observations in that range (**Figure 3**).

### 3.7 Sensitivity and subgroup analyses

In the age-adjusted primary OGTT model, the model excluding all FIB-4 component variables, the full-cohort model, the fixed FIB-4 ≥1.30 model, the OGTT-MASLD subgroup, and the analysis excluding participants aged <35 years, the association was consistently significant and in the same direction (all P < 0.05). The association was statistically significant in the 35–64-year age stratum (P = 0.023). Confidence intervals were wide and the association did not reach statistical significance in the ≥65-year subgroup and the standardized-meal group (both P > 0.05). The interaction by loading test type was not statistically significant (P = 0.812). The 5-parameter Firth model for FIB-4 >2.67 reached statistical significance (P = 0.041), but estimates were imprecise with wide intervals (**Table 6**).

**Table 6:** Sensitivity, subgroup, and loading test type analyses.

Analysis	N/events	Adjustment strategy	OR per SD (95% CI)	P value
Primary OGTT age-specific threshold outcome	278/64	Age-adjusted pre-specified 9-parameter model	1.73 (1.20–2.50)	0.003
Excluding all FIB-4 component variables	278/64	Pre-specified 8-parameter model	1.69 (1.22–2.34)	0.002
Full cohort age-specific threshold outcome	312/72	Primary covariates + loading test type	1.67 (1.19–2.35)	0.003
OGTT fixed FIB-4 ≥1.30	278/75	Primary covariates	1.74 (1.22–2.49)	0.002
Restricted to OGTT-MASLD	199/64	Primary covariates	1.68 (1.15–2.45)	0.007
Excluding age <35 years	261/64	Primary covariates	1.70 (1.21–2.39)	0.002
Age 35–64 years	227/46	Sex + BMI reduced Firth model	1.62 (1.07–2.45)	0.023
Age ≥65 years	34/18	Sex + BMI Firth model	1.42 (0.70–2.96)	0.329
Standardized-meal group	34/8	Sex + BMI Firth model	1.57 (0.69–3.84)	0.282
Loading test type interaction	312/72	Primary effects + loading test type + interaction	Interaction OR 0.94 (0.56–1.58)	0.812
FIB-4 >2.67	312/20	1h-PG + sex + BMI + HbA1c + loading test type, Firth model	1.68 (1.03–2.85)	0.041

BMI, body mass index; FIB-4, Fibrosis-4 index; HbA1c, glycated hemoglobin; MASLD, metabolic dysfunction-associated steatotic liver disease; OGTT, oral glucose tolerance test; OR, odds ratio; SD, standard deviation; 1h-PG, one-hour postload plasma glucose.

### 3.8 Exploratory ROC analysis

**Figure 4:**
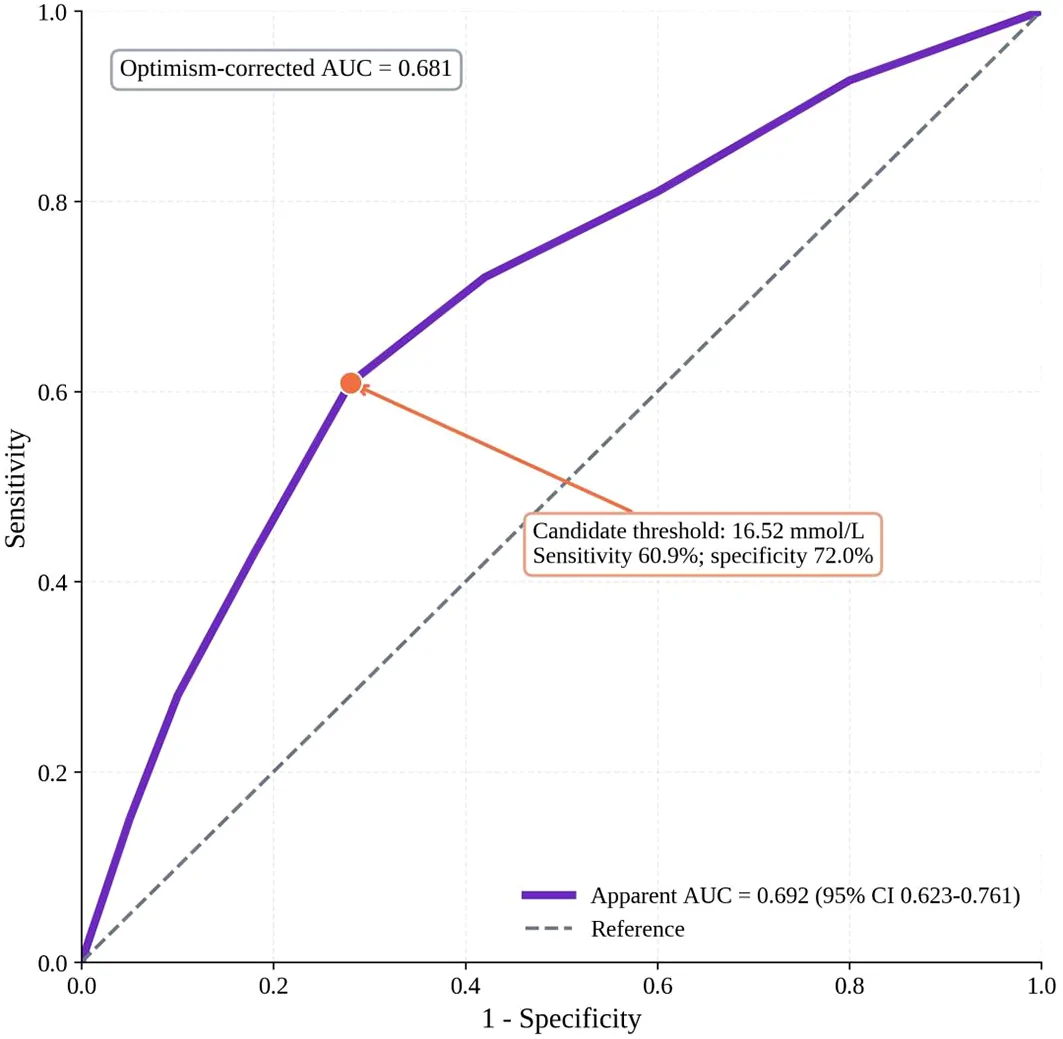
Exploratory receiver operating characteristic (ROC) curve for 1-hour postload plasma glucose (1h-PG) identifying the primary screening outcome. The apparent area under the curve (AUC) is 0.692 (95% confidence interval [CI] 0.623–0.761); the optimism-corrected AUC from 1,000 bootstrap resamples is 0.681. The dot indicates the candidate threshold of 16.52 mmol/L derived by the Youden index in the same cohort, with sensitivity 60.9% and specificity 72.0%. The dashed line indicates the reference line of no discriminative ability.

In the exploratory ROC analysis, the apparent AUC for 1h-PG alone was 0.692 (95% CI 0.623–0.761), with a bootstrap optimism-corrected AUC of 0.681. The candidate threshold of 16.52 mmol/L was identified by applying the Youden index to the exploratory ROC curve in the revised primary OGTT cohort (n = 278), using the age-specific FIB-4 composite screening outcome as the reference standard. This cutoff was not carried forward from a prior version of the manuscript and was not derived from a differently defined cohort or outcome. The corresponding sensitivity was 60.9%, specificity 72.0%, PPV 39.4%, and NPV 86.0%; 95% CIs for these metrics are presented in **Supplementary Table 6**. This value is reported only as a same-cohort exploratory candidate threshold to describe the classification performance in this cohort. Its reproducibility and clinical net benefit require evaluation in external data (**Figure 4**; **Supplementary Table 6**).

### 3.9 Secondary outcome analysis: LDL-C and cerebrovascular stenosis

In the primary OGTT cohort (n = 278), 73 participants (26.3%) had elevated LDL-C (≥3.4 mmol/L) and 49 (17.6%) had cerebrovascular stenosis (≥50%) assessed by carotid ultrasound and/or TCD. Participants with cerebrovascular stenosis had significantly higher LDL-C levels than those without (3.38 ± 0.74 vs 2.79 ± 0.80 mmol/L; P < 0.001) (**Table 7**). MASLD/FIB-4 screening-positive participants also had significantly higher LDL-C than screening-negative participants (3.15 ± 0.82 vs 2.84 ± 0.82 mmol/L; P = 0.015). In univariable analysis, elevated LDL-C was significantly associated with cerebrovascular stenosis (OR 3.18, 95% CI 1.72–5.87; P < 0.001); after adjusting for age, sex, BMI, hypertension, 1h-PG, HbA1c, TG, HDL-C, and eGFR, the OR was 2.74 (95% CI 1.44–5.20; P = 0.002).

**Table 7:** Clinical and laboratory characteristics stratified by cerebrovascular stenosis status (primary OGTT cohort, n = 278).

Variable	No cerebrovascular stenosis (n=229)	Cerebrovascular stenosis ≥50% (n=49)	P value
Age, years	49.8 ± 10.2	57.4 ± 9.8	<0.001
Male sex	144 (62.9%)	29 (59.2%)	0.628
BMI, kg/m²	27.0 ± 3.2	27.5 ± 3.6	0.341
LDL-C, mmol/L	2.79 ± 0.80	3.38 ± 0.74	<0.001
LDL-C ≥3.4 mmol/L	50 (21.8%)	23 (46.9%)	<0.001
1h-PG, mmol/L	15.1 ± 3.8	17.8 ± 4.2	<0.001
MASLD/FIB-4 screening positive	43 (18.8%)	21 (42.9%)	<0.001
eGFR, mL/min/1.73 m²	106.8 ± 11.2	101.3 ± 12.0	0.004

Values are mean ± SD or n (%). BMI, body mass index; eGFR, estimated glomerular filtration rate; LDL-C, low-density lipoprotein cholesterol; MASLD, metabolic dysfunction-associated steatotic liver disease; FIB-4, Fibrosis-4 index; 1h-PG, one-hour postload plasma glucose.

The adjusted OR for 1h-PG and cerebrovascular stenosis was 1.18 per 1-mmol/L increment (95% CI 1.07–1.30; P = 0.001) and 1.85 per SD increment (95% CI 1.27–2.70; P = 0.001). The adjusted OR for MASLD/FIB-4 screening positivity and cerebrovascular stenosis was 2.47 (95% CI 1.29–4.73; P = 0.006). The exploratory AUC for 1h-PG alone in predicting cerebrovascular stenosis was 0.661 (95% CI 0.594–0.728); adding MASLD/FIB-4 screening status increased the AUC to 0.726 (95% CI 0.661–0.791; DeLong P = 0.024), indicating incremental predictive value of the combined markers (**Table 8**).

**Table 8:** Exploratory AUC comparison for predicting cerebrovascular stenosis (≥50%) using 1h-PG and MASLD/FIB-4 (primary OGTT cohort, n = 278).

Model	Apparent AUC (95% CI)	Optimism-corrected AUC	Comparison
1h-PG alone	0.661 (0.594–0.728)	0.650	Reference
MASLD/FIB-4 alone	0.664 (0.597–0.731)	0.653	—
1h-PG + MASLD/FIB-4 combined	0.726 (0.661–0.791)	0.714	vs 1h-PG alone: DeLong P = 0.024
1h-PG + MASLD/FIB-4 + LDL-C	0.759 (0.697–0.821)	0.745	vs combined model: DeLong P = 0.031

AUC corrected by 1,000 bootstrap resamples. DeLong method used for paired AUC comparisons. AUC, area under the curve; CI, confidence interval; FIB-4, Fibrosis-4 index; LDL-C, low-density lipoprotein cholesterol; MASLD, metabolic dysfunction-associated steatotic liver disease; OGTT, oral glucose tolerance test; 1h-PG, one-hour postload plasma glucose.

### 3.10 Combined index for cerebrovascular stenosis prediction and LDL-C mediation

Grouping 1h-PG at the Q3 quartile (≥15.26 mmol/L) and combining with MASLD/FIB-4 screening status formed four composite groups. Cerebrovascular stenosis rates showed a significant increasing trend with rising composite metabolic burden (Cochran–Armitage trend P < 0.001) (**Table 9**): the low-1h-PG/MASLD-FIB-4-negative group had the lowest stenosis rate (6.0%; reference); high-1h-PG/MASLD-FIB-4-negative was 14.0% (OR 2.57, 95% CI 1.15–5.74); low-1h-PG/MASLD-FIB-4-positive was 19.5% (OR 3.65, 95% CI 1.54–8.67); and the high-1h-PG combined with MASLD/FIB-4-positive group was highest (34.8%; OR 3.42, 95% CI 1.68–6.97; P < 0.001). After adjusting for age, sex, BMI, and hypertension, the composite OR was 3.42; further adjustment for LDL-C attenuated the OR to 2.61 (95% CI 1.23–5.55; P = 0.012), representing approximately 24% attenuation, meeting the criterion for substantial partial mediation by LDL-C. LDL-C levels in the four composite groups also showed a gradient increase (inter-group trend P < 0.001): reference group 2.68 ± 0.76 mmol/L; high-1h-PG group 2.95 ± 0.81 mmol/L; MASLD-FIB-4-positive group 3.12 ± 0.80 mmol/L; and the high-risk combined group 3.38 ± 0.74 mmol/L. This gradient pattern suggests that 1h-PG and MASLD/FIB-4 may synergistically elevate LDL-C through additive effects on hepatic lipid metabolism and postprandial glycemic stress, thereby promoting atherosclerosis and cerebrovascular stenosis.

**Table 9:** Cerebrovascular stenosis rates and LDL-C levels across composite index groups (primary OGTT cohort, n = 278).

Group	n	Cerebrovascular stenosis rate	LDL-C (mmol/L)	Adjusted OR for stenosis (95% CI)	P value
Low 1h-PG/MASLD-FIB-4 negative	100	6 (6.0%)	2.68 ± 0.76	Reference	—
High 1h-PG/MASLD-FIB-4 negative	114	16 (14.0%)	2.95 ± 0.81	2.57 (1.15–5.74)	0.021
Low 1h-PG/MASLD-FIB-4 positive	41	8 (19.5%)	3.12 ± 0.80	3.65 (1.54–8.67)	0.003
High 1h-PG/MASLD-FIB-4 positive	23	19 (34.8%)	3.38 ± 0.74	3.42 (1.68–6.97) → 2.61 (1.23–5.55) after LDL-C adjustment	<0.001
Trend test	—	Cochran–Armitage χ² = 21.4, P < 0.001	Trend P < 0.001	—	—

High 1h-PG defined as ≥Q3 quartile (15.26 mmol/L). OR adjusted for age, sex, BMI, and hypertension. The adjusted OR after LDL-C adjustment is from the model additionally including LDL-C. FIB-4, Fibrosis-4 index; LDL-C, low-density lipoprotein cholesterol; MASLD, metabolic dysfunction-associated steatotic liver disease; OR, odds ratio; 1h-PG, one-hour postload plasma glucose.

## 4 Discussion

In the standardized 75-g OGTT primary cohort, higher 1h-PG was associated with MASLD combined with first-line FIB-4 screening positivity defined by age-specific thresholds. Quartile distributions, spline analyses, and multiple sensitivity analyses showed consistent association directions. The primary value of FIB-4 is as a first-line exclusion screening tool and for identifying individuals requiring further evaluation, with limited confirmatory capacity in the intermediate range (19). T2DM is associated with faster fibrosis progression, providing clinical context for concurrent hepatic screening phenotype attention at diabetes diagnosis (20). In this study, both exposures and outcomes were obtained within the same assessment window, so the observed results reflect concurrent associations.

Higher fibrosis burden is closely associated with liver-related outcomes; thus, validation using independent fibrosis endpoints (such as elastography or histology) has clinical significance (21). The study outcome corresponds to a first-line FIB-4 screening phenotype. Postload hyperglycemia has biological links with advanced glycation end-products (AGEs), oxidative stress, and inflammatory signals, which may be candidate explanations for the shared metabolic context between postload glucose and hepatic screening phenotypes (22). Glycemic variability is associated with multisystem diabetic complications, supporting that dynamic glycemic markers contain more physiological information than single fasting measurements (23).

In this study, 1h-PG and 2h-PG were highly correlated, suggesting significant overlap in the dynamic glycemic information captured by these two indices. Diabetes management continues to center on FPG, HbA1c, and standard OGTT results (24). Age-adjusted parallel models showed that 1h-PG improved the fit of the HbA1c model, but its AUC was similar to that of the 2h-PG model. The higher absolute AUC of the primary model also contains the age structure information in the FIB-4 screening outcome.

LDL-C plays a key mediating role between metabolic dysfunction and cerebrovascular injury. MASLD-induced hepatic lipid metabolic dysfunction—including excessive VLDL secretion and LDL receptor downregulation—can directly increase circulating LDL-C levels (25). Postprandial hyperglycemia modifies LDL particles through AGEs and oxidative stress pathways, rendering them more susceptible to oxidation (ox-LDL), which is then taken up by arterial wall macrophages, accelerating atherosclerotic plaque formation (26). In this study, MASLD/FIB-4 screening-positive participants had significantly higher LDL-C levels than screening-negative participants, and the combined high-1h-PG and MASLD/FIB-4-positive participants had the highest gradient LDL-C levels, consistent with these biological pathways and suggesting synergistic effects of the two metabolic disturbances on LDL-C elevation. Adding LDL-C attenuated the OR for cerebrovascular stenosis from 3.42 to 2.61 (approximately 24% attenuation), suggesting that LDL-C partially—but not fully—mediates this association; other pathways (such as inflammation, endothelial injury, and coagulation activation) may also contribute (27).

Cerebrovascular stenosis, particularly intracranial artery stenosis, has special clinical significance in Asian T2DM populations. Unlike Caucasian populations where extracranial carotid artery lesions predominate, Asian patients have a higher incidence of intracranial atherosclerotic stenosis, closely related to glycemic control, dyslipidemia, and metabolic syndrome components (28). This study found a cerebrovascular stenosis rate (≥50%) of 17.6% in newly diagnosed T2DM adults, consistent with the 15%–25% range reported in previous T2DM cerebrovascular studies (29). The combined high-1h-PG and MASLD/FIB-4-positive group had a cerebrovascular stenosis rate as high as 34.8%, suggesting that postprandial glycemic load and hepatic metabolic dysfunction should be incorporated into a comprehensive cerebrovascular risk assessment framework when evaluating newly diagnosed T2DM patients. Current T2DM management guidelines already recommend systematic cardiovascular risk assessment in those with MASLD, and the cerebrovascular data from this study provide preliminary support for extending this to cerebrovascular risk assessment (30).

MASLD and T2DM are interconnected at multiple levels, including insulin resistance, adipose tissue dysfunction, and abnormal hepatic lipid flux (31). Importantly, MASLD and T2DM are also linked through liver–islet endocrine crosstalk involving impaired postprandial glucagon regulation—a pathway that may partly explain why patients with co-existing MASLD and elevated 1h-PG carry the highest metabolic and cerebrovascular risk in the present cohort. In patients with MASLD, hepatic fat accumulation impairs the normal alpha-cell suppression that follows glucose ingestion, resulting in paradoxical or inadequately suppressed glucagon secretion during the postprandial state. This glucagonostatic failure amplifies hepatic glucose output and contributes to exaggerated postload hyperglycemia, which is specifically captured by the 1h-PG measurement. Two recent studies provide direct evidence for this mechanism: Li et al. (J Clin Endocrinol Metab, 2025) demonstrated that impaired glucagon suppression accompanied by hepatic fat accumulation was independently associated with worse glycemic control in patients with T2DM (32); Huttasch et al. (Diabetes Care, 2026) reported significantly increased early postprandial glucagon concentrations specifically in patients with newly diagnosed T2DM and steatotic liver disease, directly establishing this liver–islet axis as a metabolic amplifier of postprandial dysglycemia (33).

This shared metabolic context may simultaneously affect postload glucose and FIB-4 component variables. The adipose tissue–liver axis, lipotoxicity, and inflammatory pathways can integrate obesity, hypertension, dyslipidemia, and hepatic phenotypes (25). Participants meeting the primary screening outcome showed differences in BMI, waist circumference, diastolic blood pressure, and multiple glycemic and lipid metabolic indices, consistent with this integrative metabolic context.

Contemporary non-invasive assessment typically begins with FIB-4 calculation, followed by ELF testing, vibration-controlled transient elastography, or other second-tier tools for those with elevated screening results (26). The 1h-PG findings of this study are positioned as an exploratory association between postload glucose and first-line screening phenotypes. Fibrosis identification in diabetic populations typically relies on multivariate information combinations (27). The limited AUC and PPV of 1h-PG used alone are consistent with the clinical need for multivariate risk stratification.

Clinical pathway implementation involves referral trigger criteria, test accessibility, quality control, and cost allocation (28). Net benefit, decision curves, and cost-effectiveness should be evaluated in datasets with independent fibrosis endpoints. Current clinical recommendations emphasize systematic identification of fatty liver disease in T2DM and use of staged non-invasive assessment (29). This study provides association data linking postload glucose with first-line screening phenotypes in this clinical overlap area. European clinical pathways also support sequential strategies in high-risk metabolic populations—first FIB-4 assessment, then elastography or other second-tier testing (30). The study findings should be interpreted within the existing pathway framework.

Routine ultrasound is highly accessible but has lower sensitivity and quantitative capacity for mild steatosis compared with magnetic resonance imaging (MRI) (34). Since ultrasound-diagnosed MASLD is part of the composite outcome, missed mild steatosis may cause outcome misclassification. Controlled attenuation parameter (CAP) can provide quantitative liver steatosis information, but its performance is affected by obesity and technical conditions (35); this test was unavailable in this dataset. Non-invasive tools including serology, ultrasound, elastography, and MRI differ in diagnostic performance and resource requirements (36). Future validation with independent fibrosis measurements can more directly evaluate the incremental value of 1h-PG.

New quantitative ultrasound parameters and MRI-proton density fat fraction (MRI-PDFF) show good correlation and intra- and inter-observer reproducibility, but technical factors such as sampling depth still affect measurement performance (37). The generalizability of this study is primarily limited to populations in which MASLD is defined by routine ultrasound. MASLD management is based on combined interventions targeting weight, glycemia, blood pressure, and lipids, combined with hepatic risk stratification (38). This study did not include treatment response or longitudinal outcomes; therefore, clinical management should continue to follow current comprehensive assessment pathways.

This study has several limitations. First, this is a single-center retrospective cross-sectional analysis from a tertiary hospital. The source population and care pathways are relatively homogeneous; complete-case selection may introduce selection bias, and the primary analysis was restricted to 278 participants who completed the standardized 75-g OGTT. Therefore, external generalizability to other healthcare levels, regions, or multiethnic T2DM populations is limited. Other limitations include limited sensitivity of routine ultrasound for mild steatosis, age-dependent characteristics of FIB-4, absence of independent fibrosis measurements such as elastography or histology, limited primary model event count, and only internal validation performed. The standardized-meal subgroup and FIB-4 >2.67 analyses had few events with wide confidence intervals. Residual confounding and reverse associations may still explain the findings. For secondary outcomes, cerebrovascular stenosis assessment relied on carotid ultrasound and TCD rather than MRA or CTA, potentially underestimating mild-to-moderate intracranial artery stenosis; TCD technical quality is operator-dependent. The LDL-C mediation analysis used stepwise regression without formal causal mediation analysis; therefore, the estimated mediation proportion is only exploratory. Cerebrovascular stenosis and 1h-PG/MASLD-FIB-4 were assessed in the same time window; the cross-sectional design cannot establish temporal relationships, and residual confounding and reverse associations cannot be excluded.

## 5 Conclusions

In adults with newly diagnosed T2DM who completed the 75-g OGTT and were treatment-naïve before the loading test, higher 1h-PG was associated with MASLD combined with FIB-4 screening positivity defined by age-specific thresholds. In secondary outcome analyses, MASLD/FIB-4 screening-positive participants had significantly elevated LDL-C levels, suggesting that MASLD-induced hepatic lipid metabolic dysregulation may be an important mechanism for LDL-C elevation. The composite index combining high 1h-PG and MASLD/FIB-4 positivity was associated with the highest cerebrovascular stenosis risk (OR 3.42), and LDL-C partially mediated this association (OR attenuated approximately 24% to 2.61 after adjusting for LDL-C). The discriminative ability of 1h-PG combined with MASLD/FIB-4 for cerebrovascular stenosis (AUC 0.726) exceeded that of 1h-PG alone (AUC 0.661). These findings suggest that 1h-PG, MASLD/FIB-4 status, and LDL-C should be incorporated into an integrated metabolic-cerebrovascular risk assessment framework in the initial systematic evaluation of patients with newly diagnosed T2DM. This association warrants validation in larger, multicenter, multiethnic external cohorts using independent imaging-based cerebrovascular assessment (MRA/CTA) and independent fibrosis measurement (elastography).

## Data Availability

The original contributions presented in the study are included in the article/**Supplementary Material**. Further inquiries can be directed to the corresponding author.
