# Supplementary material for: Association of one-hour postload plasma glucose combined with metabolic dysfunction-associated steatotic liver disease and FIB-4 screening positivity with elevated LDL cholesterol and cerebrovascular stenosis
Source: Front Endocrinol (Lausanne). 2026 Aug 14;17:1887611. doi: 10.3389/fendo.2026.1887611 (PMC13521856; doi:10.3389/fendo.2026.1887611)
Supplement: Supplementary file 1 [file Table1.docx]

**Supplementary Material**

**Supplementary Table 1. Participant exclusion reasons, missingness, and distribution of glucose-lowering treatment before testing**

| **Stage/category** | **Reason or medication** | ***n*** | **Supplementary details** |
| --- | --- | --- | --- |
| Screening exclusion | Non-T2DM/special diabetes type | 8 | Counted mutually exclusively according to the first applicable reason |
| Screening exclusion | Chronic liver disease | 19 |  |
| Screening exclusion | Excess alcohol intake | 15 |  |
| Screening exclusion | Severe comorbidity | 12 |  |
| Screening exclusion | Relevant medications affecting hepatic fat or glucose metabolism | 11 |  |
| Screening exclusion | Glucose-lowering treatment before testing | 18 | See medication distribution below |
| Screening exclusion | No eligible 1 h-PG sample | 70 | Including no test record |
| Screening exclusion | 60-minute sample outside ± 10 minutes | 16 |  |
| Screening exclusion | No concurrent liver ultrasonography | 34 |  |
| Screening exclusion | Missing FIB-4 components | 18 |  |
| Screening exclusion | Missing prespecified covariates | 12 |  |
| Pretest treatment distribution | Metformin | 9 | Duration 7 [3, 12] d; interval from last dose to testing 24 [12, 36] h |
| Pretest treatment distribution | Insulin | 5 | Duration 3 [1, 5] d; interval from last dose to testing 10 [6, 14] h; acute use for symptomatic hyperglycemia in 3 participants |
| Pretest treatment distribution | Sulfonylureas | 2 | Duration 5 d and 8 d; interval from last dose to testing 24 h and 36 h |
| Pretest treatment distribution | DPP-4 inhibitors | 2 | Duration 4 d and 7 d; interval from last dose to testing was 24 h for both |
| Final analysis set | Any glucose-lowering medication or insulin | 0/312 | No glucose-lowering treatment before load testing |

Note: *n* indicates number of participants, d indicates days, and h indicates hours; values in brackets are interquartile ranges (IQRs). Exclusion reasons were counted mutually exclusively according to the first applicable reason. The 18 participants who received glucose-lowering treatment before testing were all excluded during screening. This table presents descriptive flow information, and no hypothesis tests were performed; therefore, no *P* values are reported. T2DM, type 2 diabetes mellitus; 1 h-PG, 1-hour postload plasma glucose; FIB-4, fibrosis-4 index; DPP-4, dipeptidyl peptidase-4.

**Supplementary Table 2. Selective comparison of available characteristics between included and excluded participants**

| **Comparable variable** | **Included participants (*n* = 312)** | **Excluded participants (*n* = 233)** | ***P* value** |
| --- | --- | --- | --- |
| Age, years | 51.6 ± 10.6 | 52.0 ± 11.2 | 0.673 |
| Male | 189 (60.6%) | 137 (58.8%) | 0.675 |
| BMI, kg/m² | 27.3 ± 3.3 | 27.0 ± 3.5 (available *n* = 211) | 0.321 |

Note: *n* indicates number of participants. Continuous variables are presented as mean ± standard deviation (SD); the independent-samples *t* test was used for approximately normal data with equal variances, and the Welch *t* test was used when variances were unequal. Categorical variables are presented as number (percentage), and the Pearson *χ²* test was used to compare proportions between groups. *P* values are two-sided. BMI was available for only 211 excluded participants. This limited comparison of available variables describes observable characteristics only and cannot exclude unmeasured selection factors. BMI, body mass index.

**Supplementary Table 3. Correlations of 1 h-PG with major metabolic and FIB-4 indicators**

| **Variable** | **Method** | **Correlation coefficient (*r* or *ρ*)** | ***P* value** |
| --- | --- | --- | --- |
| FIB-4 | Spearman | 0.301 | < 0.001 |
| FPG | Pearson | 0.144 | 0.016 |
| 2 h-PG | Pearson | 0.857 | < 0.001 |
| HbA1c | Pearson | 0.218 | < 0.001 |
| AST | Spearman | 0.188 | 0.002 |
| Platelet count | Pearson | -0.149 | 0.013 |
| BMI | Pearson | -0.004 | 0.949 |
| TG | Spearman | 0.065 | 0.278 |

Note: *n* = 278. The Pearson correlation coefficient *r* measures the linear association between two continuous variables, whereas the Spearman rank correlation coefficient *ρ* measures rank-based monotonic association; both range from -1 to 1. *P* values are two-sided tests of whether the corresponding correlation coefficient equals 0. 1 h-PG and 2 h-PG were strongly correlated and therefore were not entered simultaneously in the same parallel model. 1 h-PG/2 h-PG, 1-/2-hour postload plasma glucose; FPG, fasting plasma glucose; HbA1c, glycated hemoglobin; AST, aspartate aminotransferase; BMI, body mass index; TG, triglycerides; FIB-4, fibrosis-4 index.

**Supplementary Table 4. Distribution of the primary outcome across 1 h-PG quartiles**

| **Quartile** | **Range, mmol/L** | ***n*** | **Outcome events** | **Proportion (95% CI)** |
| --- | --- | --- | --- | --- |
| Q1 | ≤ 12.50 | 70 | 5 | 7.1% (3.1%-15.7%) |
| Q2 | 12.51-15.25 | 70 | 11 | 15.7% (9.0%-26.0%) |
| Q3 | 15.26-18.35 | 69 | 18 | 26.1% (17.2%-37.5%) |
| Q4 | > 18.35 | 69 | 30 | 43.5% (32.4%-55.2%) |

Note: *n* indicates number of participants, and the outcome proportion was calculated as events/*n*. The 95% confidence interval (CI) used the binomial Wilson score interval. The Pearson *χ²* test was used to compare overall proportions across the four quartiles, and the Cochran-Armitage trend test was used to evaluate a linear trend across increasing quartile ranks. *P* values are two-sided. Between-group *χ²* = 28.73 and trend *χ²* = 27.91; both *P* < 0.001. Q1-Q4 indicate quartiles from low to high. 1 h-PG, 1-hour postload plasma glucose; MASLD, metabolic dysfunction-associated steatotic liver disease; FIB-4, fibrosis-4 index. The primary screening outcome was defined as ultrasonographic MASLD with FIB-4 meeting age-specific thresholds.

**Supplementary Table 5. Bootstrap internal validation of the main model**

| **Internal validation metric** | **Value** | **Explanation** |
| --- | --- | --- |
| Events/parameters | 64/9 | Events-per-parameter ratio = 7.1 |
| Maximum VIF | 1.88 | No obvious multicollinearity |
| Apparent AUC | 0.801 | Absolute discrimination includes age-structure information |
| Mean AUC optimism | 0.015 | 1,000 bootstrap resamples |
| Optimism-corrected AUC | 0.786 |  |
| Apparent Nagelkerke *R²* | 0.383 |  |
| Optimism-corrected Nagelkerke *R²* | 0.339 |  |
| Calibration slope | 0.91 | Mild overfitting |
| Uniform shrinkage factor | 0.91 | Shrunken 1 h-PG OR approximately 1.65 |

Note: Bootstrap validation used 1,000 resamples. In each resample, the model was fitted and performance was evaluated in the resample and the original sample to estimate mean optimism and the optimism-corrected value. Events/parameters and the events-per-parameter ratio describe model complexity. VIF indicates variance inflation factor and was used to assess multicollinearity. ROC indicates receiver operating characteristic curve, and AUC indicates area under the ROC curve. Nagelkerke *R²* is a pseudo coefficient of determination for the logistic model. Calibration slope measures the agreement between predicted effect strength and observed outcomes, with an ideal value of 1. The uniform shrinkage factor is used to shrink regression coefficients by the same proportion to reduce overfitting. OR, odds ratio; 1 h-PG, 1-hour postload plasma glucose.

**Supplementary Table 6. Exploratory ROC candidate cutoff and performance intervals**

| **Metric** | **Estimate** | **95% CI** | **Calculation basis** |
| --- | --- | --- | --- |
| AUC | 0.692 | 0.623-0.761 | DeLong |
| Bootstrap-corrected AUC | 0.681 | — | 1,000 bootstrap resamples |
| Candidate cutoff | 16.52 mmol/L | — | Same-cohort Youden index |
| Sensitivity | 60.9% | 48.7%-71.9% | 39/64, Wilson |
| Specificity | 72.0% | 65.6%-77.6% | 154/214, Wilson |
| PPV | 39.4% | 30.3%-49.2% | 39/99, Wilson |
| NPV | 86.0% | 80.2%-90.4% | 154/179, Wilson |

Note: ROC indicates receiver operating characteristic curve, and AUC indicates area under the ROC curve. The 95% confidence interval (CI) for AUC used the nonparametric DeLong method. The candidate cutoff was determined using the Youden index (sensitivity + specificity - 1) in the same cohort. Sensitivity is the proportion of outcome-positive participants who tested positive, and specificity is the proportion of outcome-negative participants who tested negative. PPV indicates positive predictive value, the proportion of outcome-positive participants among those who tested positive. NPV indicates negative predictive value, the proportion of outcome-negative participants among those who tested negative. The 95% CIs for these proportions used Wilson score intervals. PPV and NPV depend on the outcome proportion in this study. — indicates that no interval was estimated. 1 h-PG, 1-hour postload plasma glucose; MASLD, metabolic dysfunction-associated steatotic liver disease; FIB-4, fibrosis-4 index. The primary screening outcome was defined as ultrasonographic MASLD with FIB-4 meeting age-specific thresholds.
